# A Novel Surgical Technique for the First Case of Neglected Bilateral Severe Congenital Genu Recurvatum Combined with Bilateral Talipes Equinocavovarus

**DOI:** 10.3390/medicina61030543

**Published:** 2025-03-20

**Authors:** Kuan-Lin Liu, Ting-Yu Hung, Xin-Le Eng, Ing-Ho Chen

**Affiliations:** 1 Department of Orthopedics, Hualien Tzu Chi Hospital, Buddhist Tzu Chi Medical Foundation, Hualien 970473, Taiwan; liulob@gmail.com (K.-L.L.); hungtingyu1021@gmail.com (T.-Y.H.); 2 School of Medicine, Tzu Chi University, Hualien 970374, Taiwan; 3 Sports Medicine Center, Hualien Tzu Chi Hospital, Buddhist Tzu Chi Medical Foundation, Hualien 970473, Taiwan; 4 Medical Department, Hualien Tzu Chi Hospital, Buddhist Tzu Chi Medical Foundation, Hualien 970473, Taiwan; engxinle@gmail.com

**Keywords:** congenital genu recurvatum, bilateral talipes equinocavovarus, novel surgical technique

## Abstract

Congenital genu recurvatum (CGR) is a rare congenital knee deformity that can be diagnosed pre- or postnatally. Most CGR cases are treated during infancy by manipulation and serial casting or splinting. Only a few neglected cases of CGR in adults with the necessity of surgical treatment have been reported. This study presents the first case of neglected bilateral severe CGR combined with bilateral talipes equinocavovarus (BTE) in a 25-year-old woman. This case is unique, because BTE is secondary to CGR with persistent progression into adulthood. The patient underwent two-stage surgeries with 1.5-month intervals to correct the knee and foot/ankle deformities. For CGR, a novel technique that combines posterior closing and anterior open wedge osteotomy was carried out to separately address the issues of 80° and 60° deformity, achieving a total correction of 140° for both knee hyperextension deformities. For BTE, the modified Lambrinudi triple arthrodesis and almost complete plantar fascia release were performed for the treatment of foot/ankle deformities. The patient had no neurovascular problems, and the wounds healed well. After 1 year of hospital-based rehabilitation training, the patient could stand and walk without the help of any walking-assist devices. The outcomes of the three- and nine-year follow-ups were highly satisfactory. Our patient represents a technically challenging case for orthopaedic surgeons because of the complexity of the surgical plan and techniques for the treatment of a patient concurrently having CGR and BTE.

## 1. Introduction

Congenital genu recurvatum (CGR) is a rare congenital knee deformity characterised by hyperextension of the knee greater than 0°, which is associated with limited flexion, and this condition is characterised by the prominence of femoral condyles in the popliteal fossa and increased transverse skin folds over the anterior surface of the knee [1]. CGR has an incidence of 1 per 100,000 live births with unknown aetiology [2]. CGR can be unilateral or bilateral and may occur as an isolated malformation or be associated with other orthopaedic abnormalities [1]. CGR can be diagnosed pre- or postnatally [2,3,4], and most of the cases are treated during infancy by manipulation and serial casting or splinting without surgical intervention [4,5,6,7,8,9,10,11,12]. So far, only a few neglected cases of CGR in adults with posterior closing wedge osteotomy have been reported [13,14,15]. When patients are neglected, their daily activities and locomotion may be impeded, leading to other orthopaedic complications towards adolescence. This study presents the first case of neglected bilateral severe CGR combined with bilateral talipes equinocavovarus in a 25-year-old woman. The details of our novel surgical technique and the long-term outcome for this patient are described herein.

## 2. Case Report

### 2.1. History and Presentation

**Figure 1:**
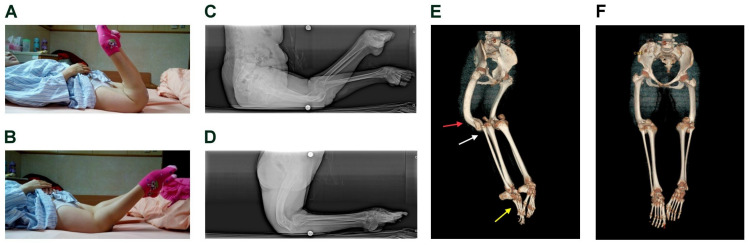
Pre-operative clinical photos and radiologic images. Photographs of the patient while lying flat on the bed show the recurvatum (hyperextension) deformity of 130° of both knees in the extension position (**A**) and recurvatum of 40° of both knees in the flexion position (**B**). The triple films show the patient using her buttock and posterior thigh to sit (**C**) and the popliteal fossa and calf to stand and walk (**D**). Pelvic to lower limb 3D-CT reconstruction (**E**) shows both distal femur shafts anterior bowing (red arrow) with femoral condyle hypoplasia and tibial anterior subluxation (white arrow). Additionally, both feet present as talipes equinocavovarus (yellow arrow). An anterior-posterior image of pelvic to lower limb 3D-CT reconstruction is also shown (**F**).

A 25-year-old lady with normal cognition and mental status was referred to our outpatient department because of severe bilateral knee and foot deformities (Figure 1). Although the knee abnormalities were detected immediately after birth, they were not treated right away. The hyper-extensive deformity of both knees was approximately 130° during extension and 40° during flexion (Figure 1A,B). The patient could use her normal hip and buttock to sit (Figure 1C) and use her both popliteal fossa and calves to touch the ground as the feet and support her body weight when walking (Figure 1D). Due to the lack of use of the calf muscles and weight loading on foot, the Achilles tendon and plantar fascia were tight and shortened. Consequently, the ankle and foot became fixed in ankle plantar flexion and talipes deformity (Figure 1E). No other physical disease, systemic disease, neurological deficits, or other associated congenital anomalies were noted. No similar symptoms were observed among her family members, and her vaccination was scheduled after birth.

### 2.2. Physical Examination and Pre-Operative Image Findings

**Figure 2:**
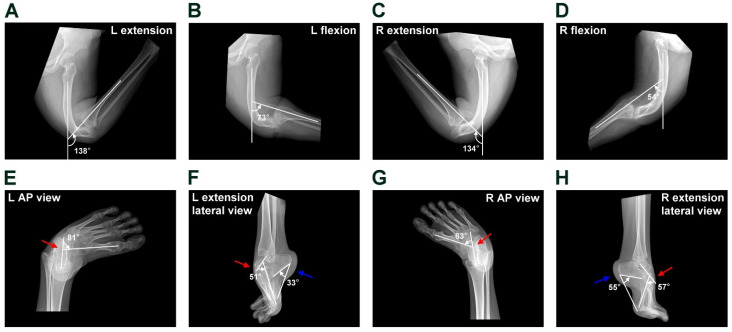
Pre-operative radiograph of the knees and feet. Both knee dynamic x-rays show the recurvatum deformity with various angles in extension and flexion (**A**–**D**). The foot and ankle anteroposterior (AP) and extended lateral view show talipes equinocavovarus with various angles of AP Meary’s angle (red arrow), lateral Meary’s angle (red arrow) and Calcaneal pitch (blue arrow) (**E**–**H**). The angles are indicated in the images.

Physical examination revealed bilateral knee recurvatum. The left knee’s range of motion (ROM) was 65° (extension, −138°; flexion, −73°), and the right knee’s ROM was 80° (extension, −134°; flexion, −54°). The hip motion was normal. The quadriceps were tight and atrophied. The patellar was small and could not engage during knee flexion. The knee varus and valgus stability were good. Keratoderma was observed over the popliteal area. The below-knee neurovascular function was intact. As shown in Figure 1A,B, no crossing of the legs was observed during extension and flexion, suggesting no varus–valgus deformity. Also, 3D-CT (Figure 1F) showed no joint line obliquity and leg length discrepancy. Both ankles were fixed in plantar flexion (Figure 1E). The feet presented as equinocavovarus deformity. The radiological images show distal femoral shaft recurvatum deformity, knee joint subluxation and patella femoral joint subluxation with various angles of extension and flexion (Figure 2A–D). The feet and ankles’ radiological images show the talipes equinocavovarus with various angles of anteroposterior (AP) and lateral Meary’s angle and Calcaneal pitch (Figure 2E–H).

### 2.3. A Novel Surgical Technique for CGR

**Figure 3:**
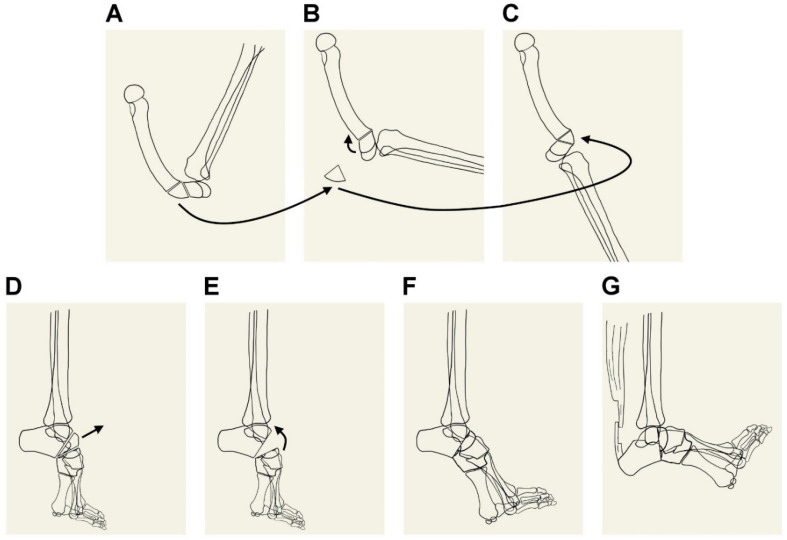
Schematic presentation of a novel distal femur osteotomy technique (**A**–**C**) and complicated foot/ankle surgical procedures (**D**–**G**). Posterior closing wedge osteotomy is performed by removing the 80° wedged bone (**A**,**B**). The posterior closing area is fixed temporarily by using screws and wire to create a rotational hinge. Then, the knee is flexed to open the anterior part of the distal femur. An appropriate triangular bone block of approximately 60° at the tip is inserted in this area (**C**) to achieve 140° correction. These surgical procedures are indicated by the arrows in panels (**A**–**C**). The mid-foot modified Lambrinudi triple arthrodesis is performed by removing the naviculum and talar head and extensive osteotomy to the anterior process of the calcaneus combined with plantar fascia release (**D**–**F**). These surgical procedures are indicated by the arrows in panels (**D**,**E**). The ankle flexion contracture is treated by Achilles lengthening with Z-plasty combined with allogenous tendon graft augmentation (**G**).

Considering the small bone stock and large deformity angles around the distal femur, this case underwent a novel surgical procedure. Deformities of 140° needed to be treated to achieve a straight knee when extended. Given that the deformity was severe, a novel technique that combined closing and open wedge osteotomies was introduced. Before surgery, a real-size cardboard was used to imitate the surgical situation (Figure 3A–C). A lateral approach to the distal third of the femur was performed. Firstly, a posteriorly based closing wedge osteotomy was made by removing the most deformed triangular bone to obtain 80° correction (Figure 3B). Second, the remaining 60° deformity was corrected using the anterior open procedure after temporarily fixing the posterior edge as a rotational hinge. The previously resected bone block was modified and then used to support the open space (Figure 3C). Thereafter, a Depuy–Synthes distal tibial locking plate with hand-made bending was used for fixation. Finally, a long leg cast was applied for external support.

### 2.4. Complicated Surgical Procedure for Equinocavovarus Deformity and Ankle Flexion Contracture

One and a half months after successfully correcting the knee deformity, the ankle flexion contracture and talipes equinocavovarus needed to be treated for ambulation. The mid-foot closing wedge osteotomy was applied (Figure 3D–F). Similar to modified Lambrinudi triple arthrodesis, the naviculum and talar head were removed. The calcaneocuboid joint was partially resected. The plantar fascia was released. After correction, multiple Kirschner wires were used for fixation. The fixed ankle plantar flexion was treated by Achilles lengthening via Z-plasty combined with allogenous tendon graft augmentation (Figure 3G). Finally, a short leg cast was applied for external support.

### 2.5. Post-Surgery Trimming and Training

The patient did not suffer from any surgery-related complications. The left distal femur remained a recurvatum deformity and was revised two weeks after the initial surgery. Similar to the situation in the left knee, the right distal femur was revised five months later, and the right foot was revised four months later. The patient started to receive progress weight-bearing exercises 1 month after each surgery. The patient had no neurovascular problems, and the wounds healed well. After one year of hospital-based rehabilitation training mainly for improvements in muscle strength and the joint range of motion, the patient was able to stand and walk without any walking-assist devices.

### 2.6. Outcomes of the Three- and Nine-Year Follow-Ups

**Figure 4:**
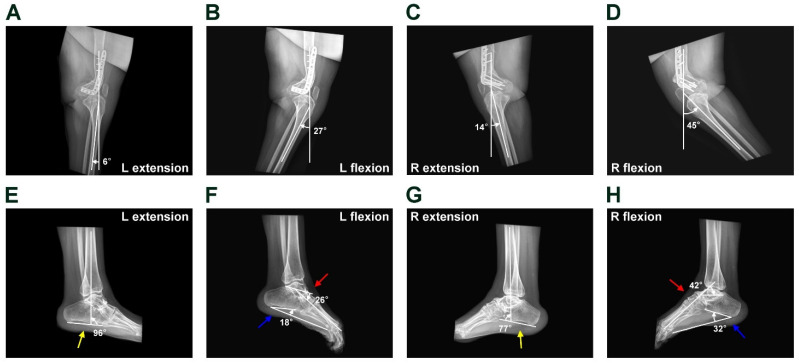
Postoperative 3-year lateral radiograph of both knees and ankle-foot dynamic films. The views show the flexion and extension angles in the left and right knees (**A**–**D**). The dynamic lateral films of the foot/ankle show the tibiocalcaneal angle (yellow arrow), Meary’s angle (red arrow) and Calcaneal pitch (blue arrow, (**E**–**H**)).

During the three-year follow-up, the patient could stand straight and walk normally without using any walking-assist devices. She could perform daily activities like healthy individuals. The knees were straight and stable. The left knee’s range of motion (ROM) was 21° (extension, 6°; flexion, 27°), and the right knee’s ROM was 31° (extension, 14°; flexion, 45°). The dynamic images of the knees are shown in Figure 4A–D. The healing of the bone at osteotomy sites was satisfactory. The postoperative degree of distal femur hyperflexion was 58° and 61° for the left and right knees, respectively. For the foot and ankle, both ankle joints were tight. Hypertrophic scars were observed over the heels. The left and right ankle motions were 6° and 1°, respectively (measured by the difference in tibiocalcaneal angle in dynamic views). The extension tibiocalcaneal angles, Meary’s angle and calcaneal pitch are shown in Figure 4E–H.

**Figure 5:**
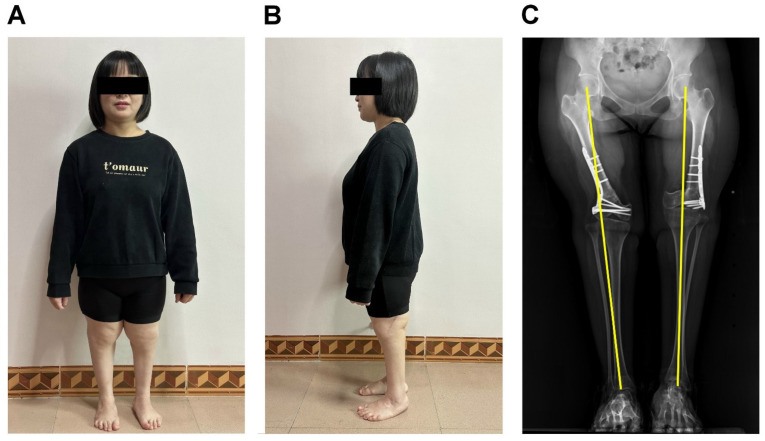
Postoperative 9-year photograph and radiography of the patient in the standing position. The front (**A**) and lateral photograph (**B**) show normal standing posture. The anterior–posterior triple film shows good alignment of the mechanical axis (yellow line) (**C**).

At the nine-year follow-up, the outcome of the patient remained highly satisfactory. The knees were straight in front and lateral views (Figure 5A,B). The radiograph showed good alignment of the mechanic axis (Figure 5C). During the follow-up period, the patient did not develop any knee osteoarthritis and did not complain of knee pain. Knee physical exams showed intact varus–valgus stability. Also, the anterior drawer and posterior drawer tests were all negative. The patient got married five years after the surgery. She subsequently had two healthy full-term boys without CGR.

## 3. Discussion

This report describes the first case of neglected bilateral severe CGR combined with bilateral talipes equinocavovarus. The patient underwent a series of well-planned surgical procedures carried out by using a novel technique to correct these orthopaedic abnormalities. Following the post-surgery rehabilitation training for one year, the patients could stand and walk without using any walking-assist devices. The clinical outcomes of the three- and nine-year follow-ups for this patient were highly satisfactory.

The patient had no family history of CGR. Thus, its aetiology remains unclear. Several methods have been proposed to classify congenital knee dislocation based on neonatal clinical examination [16]. Most of the CGR cases are treated during infancy by using conservative treatments, such as manipulation and serial casting or splinting, and good outcomes are normally obtained [4,5,6,7,8,9,10,11,12]. In neglected cases, patients may develop further musculoskeletal disorders, thus requiring surgical intervention with high complexity. To date, only a few neglected cases of CGR have been reported. Some studies [13,14,15] involve the treatment of patients at the ages of 13, 16 and 43 years, with both knees showing hyperextension deformities of 90° [13], 150° [14] and 135° [15], respectively. In all these three cases, posterior closing wedge osteotomy was the major surgery that was carried out to correct the deformity. In the patient, both knee hyperextension deformities reached 140° because of the double deformity of distal femoral anterior bowing and joint anterior subluxation. A novel technique that combines posterior closing and anterior open wedge osteotomy was then introduced to separately address the issues of 80° and 60° deformity. Notably, the bone block resected during closing wedge osteotomy was used to support the opened space during open wedge osteotomy. The efficacy of this novel surgical technique is evidenced by the clinical outcomes; the patient’s knees were straight and stable in the standing position and had a good alignment of the mechanical axis.

Our novel surgical technique, which involves the combination of posterior closing and anterior open wedge osteotomy, has some benefits. Firstly, this method allows the correction of severe deformity while preserving the maximum length of the limb. By applying this surgical combination, not a piece of bone was wasted. Secondly, this procedure can preserve plenty of condylar bone stock for plate fixation, especially for short-status patients similar to the present case. Third, considering the persistent elongation of the posterior vascular structure, this novel technique could avoid soft tissue redundancy and further fatal vascular kinking as compared to posterior closing wedge osteotomy alone. Nevertheless, this procedure also has some shortcomings. Firstly, a risk of bony non-union was observed because of the large number of periosteal dissection and additional bone interface. A demineralised bone matrix on the osteotomy part was applied to the patient to enhance bone healing. Secondly, the anterior opening procedure may increase quadricep tension, resulting in a loss of the knee flexion angle. Some studies have carried out quadricep lengthening (V-Z plasty) [13,15] to address this issue. However, quadricep lengthening and anterior release were not carried out based on the concerns that these procedures may decrease quadricep power. Our patient did not necessitate quadriceps lengthening for two reasons. First, the knees of our patient were not stiff before surgery. The left and right knee’s ROM were 65° and 80°, respectively. Thus, the quadriceps muscle and tendon still had certain elasticity. Second, while performing anterior opening osteotomy, the quadriceps muscles attached to the bone were released.

Equinocavovarus deformity of the foot is often seen in patients who have congenital anomalies, poliomyelitis, neuromuscular disease, or traumatic brain injury [17]. The aetiology of CGR in our patient is unique because talipes equinocavovarus was secondary to CGR with persistent progression into adulthood. Almost all of the CGR cases reported previously [13,14,15] walked on all fours, so these patients had no foot and ankle deformities. In the present study, the patient walked using the popliteal fossa and leg as the foot. Therefore, she did not need to dorsiflex the ankle and foot. She used her leg and plantarflexed foot to resorb the impacted force while jumping. Hence, the mid-foot and fore-foot need to flex instead of the dorsiflex to accommodate these activities. As a result, both of her ankles developed fixed flexion contracture and foot equinocavovarus deformity since her teenage years. After straightening the knees of the patient, a large degree of foot/ankle correction was necessary for normal plantigrade locomotion. Several surgical techniques can be performed to treat equinocavovarus deformity of the foot [18,19]. For our patients, the modified Lambrinudi triple arthrodesis and almost complete plantar fascia release were chosen to correct foot/ankle deformities because of the expeditious correction potential of deformity. The efficacy of this surgical technique is evidenced by the clinical outcomes; the patient was able to stand and walk without any walking-assist devices and perform activities of daily living.

In our case, we had set the target post-operative outcomes for knees and feet for this patient. After surgery, we expected the knees to be straight while standing and the feet to perform bipedal locomotion while walking. However, these outcomes could not be completely achieved by one surgery due to the potential risk of vascular and skin complications. As such, the revision surgeries were performed to achieve the target post-operative outcomes and could be considered potential complications related to the deformity and surgical technique.

The current study has at least two limitations. First, this study only presented a single case and had no control case. As such, the generalisation of our findings to other similar cases is limited. Second, the nine-year follow-up was performed to assess the outcomes of our patient. Postoperative outcomes after this follow-up period remain unknown.

## 4. Conclusions

In conclusion, this study reports the first neglected case of bilateral severe CGR combined with bilateral talipes equinocavovarus who underwent two-stage surgeries to correct knee and foot/ankle deformities. The short- and long-term clinical outcomes of this case were highly satisfactory without any complications. Our patient represents a technically challenging case for orthopaedic surgeons because of the complexity of the surgical plan and techniques for the treatment of a patient concurrently having CGR and talipes equinocavovarus.

## Data Availability

All data shown in this study are included in the published article.

## Abbreviations

The following abbreviations are used in this manuscript:

CGR	congenital genu recurvatum
BTE	bilateral talipes equinocavovarus
ROM	knee’s range of motion
AP	anteroposterior
